# Estradiol as the Trigger of Sirtuin-1-Dependent Cell Signaling with a Potential Utility in Anti-Aging Therapies

**DOI:** 10.3390/ijms241813753

**Published:** 2023-09-06

**Authors:** Kamil Karolczak, Cezary Watala

**Affiliations:** Department of Haemostatic Disorders, Medical University of Lodz, ul. Mazowiecka 6/8, 92-215 Lodz, Poland; cezary.watala@umed.lodz.pl

**Keywords:** estradiol, sirtuin 1, aging

## Abstract

Aging entails the inevitable loss of the structural and functional integrity of cells and tissues during the lifetime. It is a highly hormone-dependent process; although, the exact mechanism of hormone involvement, including sex hormones, is unclear. The marked suppression of estradiol synthesis during menopause suggests that the hormone may be crucial in maintaining cell lifespan and viability in women. Recent studies also indicate that the same may be true for men. Similar anti-aging features are attributed to sirtuin 1 (SIRT1), which may possibly be linked at the molecular level with estradiol. This finding may be valuable for understanding the aging process, its regulation, and possible prevention against unhealthy aging. The following article summarizes the initial studies published in this field with a focus on age-associated diseases, like cancer, cardiovascular disease and atherogenic metabolic shift, osteoarthritis, osteoporosis, and muscle damage, as well as neurodegenerative and neuropsychiatric diseases.

## 1. Introduction

Aging is a naturally occurring process where the body gradually loses its structural integrity over time, thus increasing the overall risk of disease and reducing life quality [1]. At the cellular level, aging results in cell-specific disorders of cell cycle regulation; this affects the regenerative capacity of stem cells and the susceptibility of cells to carcinogenesis [2,3]. The body also loses the ability to regulate its metabolism in a manner adequate to the needs of cells, which is particularly visible in the metabolism regulation in mitochondria, shifting the redox balance towards the intensification of the production of reactive oxygen species, resulting in the increased oxidative damage of biomacromolecules [4] and impaired cell renewal due to impaired autophagy [5].

Changes also occur in the actions of numerous body systems, including the endocrine system, which begins to secrete hormones in a pattern that is usually significantly different from that observed in earlier stages of life. This may be due to variations in the functioning of the feedback systems responsible for the multi-stage regulation of hormone secretion. It is not entirely clear whether any of the changes in the endocrine system associated with aging may be beneficial adaptations to biochemical challenges encountered at older ages and which of them may be simple side effects present in endocrine glands affected by chronic inflammation, hyperglycemia, or other chronic disorders usually related to the later stages of ontogenesis [6].

The most noticeable feature of aging is menopause, which results in the termination of the reproductive function in the ovaries and a significant reduction in their secretory activity; although, they still remain able to produce small amounts of estradiol, estrone, and dehydroepiandrosterone. Menopause is usually accompanied by a set of symptoms that can significantly affect the quality of life and increase the risk of diseases, including those that may be life-threatening [7,8]. Among the health consequences associated with menopause, the most serious is an increased risk of depression, associated with changes in the neurotransmission networks in the brain modulated by estradiol [9,10,11], as well as neurodegeneration [12,13], osteoarthritis [14], osteoporosis [15,16], heart remodeling [17,18], weakness of the skeletal muscles [19], and atherogenic metabolic disorders [20,21].

Estrogens, such as estradiol, also play a significant role in the regulation of metabolism in men and the fall in estradiol levels observed with age [22,23]; although not as obvious as in the case of women, this can also significantly modulate the pattern of male healthy aging. About 20% of the estradiol circulating in the peripheral blood of men comes from the testes; but, the vast majority is derived from metabolism in the peripheral tissues (extragonadal steroidogenesis). It should be emphasized that the local production of estradiol in male peripheral tissues is not necessarily reflected in circulating blood estradiol levels as local and tissue estradiol may act in an autocrine or paracrine fashion at the site of synthesis, or in its immediate vicinity, without entering the bloodstream [24]. It has been found that serum estradiol correlates positively with bone mineral density in older men [25] and that estradiol protects neuroglia from neuroinflammation [26], which is a significant factor contributing to depression [27] and neurodegeneration [28]. Low estradiol levels are also associated with a higher cardiovascular risk in men [29].

Thus, estradiol appears to support the maintenance of a healthy pattern of aging, i.e., extending the period of old age free of chronic diseases of the nervous, osteoarticular, and cardiovascular systems, in women and men. Nevertheless, our understanding of the exact molecular mechanisms shaping the relationship between estradiol and aging is still far from complete.

Thus, the molecular basis of the potentially beneficial associations between healthy aging and estradiol levels remains elusive. It also remains unclear how estradiol protects the brain, heart, blood vessels, bones, and joints from stress, the intensity of which may increase with age. Furthermore, how estradiol maintains the activity of cellular protective systems, preventing them from excessive use or age-related reductions in efficiency remains unclear, as does whether these mechanisms are always beneficial and whether they may bring about surprising unwanted health outcomes.

## 2. SIRT1 and Its Mutual Interactions with Estrogen Receptors

Sirtuin 1 (SIRT1, silent mating type information regulation 2 homolog) is a NAD^+^ (nicotinamide adenine dinucleotide)-dependent deacetylase crucial for the cellular response to exercise and diet; it has demonstrated very promising abilities to extend lifespans in rodents, primates, and possibly also in humans [30]. SIRT1 stimulation appears to counteract bone loss and prevent chondrocyte degeneration [31]. Some model studies (in primary rat astrocytes [32] and animal models of depression [33,34]) suggest that SIRT1 activation may protect against neurodegeneration. SIRT1-dependent autophagy seems to protect cardiovascular tissues against inflammation, oxidative stress, and aging [35].

Thus, SIRT1 could probably be involved in the pathways associated with aging, some of which appear to overlap with anti- or pro-aging reactions stimulated by estradiol or estradiol deprivation, respectively. 

The following chapters will examine the currently available evidence of a cross-talk between estradiol and SIRT1 in cellular responses, possibly showing anti-aging properties.

The relationship between estradiol and SIRT1 expression has so far been identified in several types of cells, tissues, and organs. Estradiol has been shown to affect the expression of SIRT1 mRNA and/or proteins in the brain (both in experimental rodents [36,37,38] and in vitro in the SH-SY54 (neuroblastoma cell line)) [36]; heart (in experimental animals in vivo) [39,40]; skeletal muscles (in vivo in animal models and in the C2C12 myoblast cell line) [41]; aorta (in vivo in animal models) [42]; vascular smooth muscle (isolated from diabetic rats) [43]; vascular endothelium (in human umbilical vein endothelial cell (HUEVEC) and human aortic endothelial cell (HAEC) cell lines) [44,45]; in vitro, in systems using Hep3B (hepatoma cell line) and HEK293 (human embryonic kidney cell) cell lines [46,47], and in vivo, in mice liver [48]; lung cancer cells (A549 and H14350 (lung cancer cell lines)) [49]; cervical cancer cells (HeLa cell line) [50] and mammary gland cancer cells using different cell lines, like MCF-7, T47D, MDA-MB-231, and SkBr3 [51,52]; peripheral mononuclear blood cells isolated from [53] mouse bone marrow [54]; and chondrocytes (ATDC5 cell line) [55]. As can be seen, few tissues appear to exhibit any relationship between estradiol and SIRT1 expression and the quite short list of tissues tested to this date suggests that this field is in the initial phase of investigations. A quick glance at the above list shows that such research is focused on tissues for which the risk of disease increases with age, many of which tend to be triggered by menopause; thus, they can be possibly associated with estradiol-dependent cellular pathways. There are also data relating to conditions found in younger populations rather than peri- or post-menopausal women, such as brain damage associated with alcohol intoxication during fetal development [37], which clearly indicate the connection between estradiol and SIRT1.

In the classical scenario, estradiol binds to one of its membrane receptors, i.e., estrogen receptor-alpha or beta (ERα or ERβ), and triggers a cascade of intracellular events leading to the activation of estrogen-responsive genes. The crucial feature of estradiol is its ability to increase the expression of its receptors, for example, the expression of ERβ in non-small-cell lung cancer cells is augmented after estradiol stimulation [49]. 

Estradiol can increase the expression of the SIRT1 protein in many types of cells, including the brain in animal models and in vitro cultured cell lines [36,37,38], heart [39,40] and skeletal muscles cultured in vitro [41], aorta in vivo in an animal models [42], vascular smooth muscles isolated from animals and treated in vitro [43] and vascular endothelium in vitro [44,45], hepatocytes in vivo in animal models [48], lung cancer cells [49], cervical cancer cells (HeLa cell line) [50] and mammary gland cancer cells (different cell lines mentioned throughout the review) [51,52], bone marrow in mouse animal models [54], and chondrocytes (ATDC5 cell line) [55]. 

It is possible that mutual increases (i.e., increases in expression of estrogen receptors α and β and SIRT1 in some cells) are interconnected. After the binding of its ligand, the estrogen receptor can directly interact with the SIRT1 molecule, which, in turn, can co-activate the estrogen receptor. Thus, a positive feedback mechanism exists between the estrogen receptor and SIRT1. SIRT1 is involved in the classical regulatory interactions in the cell nucleus between a transcription factor, like an estrogen receptor, and its substrates—SIRT1 in this case, which immediately becomes an activator of the receptor. Interestingly, the deacetylating activity of SIRT1 is not necessary for the mentioned feedback loop. Since the SIRT1 molecule can undergo an autonomous, intrinsic autoinhibition, the self-regulatory mechanism inherent in SIRT1 may also influence the expression of the estrogen receptor [47]. 

Thus, the estradiol-activated estrogen receptor significantly affects SIRT1 expression, which, in turn, modulates the expression of the estrogen receptor. Therefore, SIRT1 can be regarded as a “mini-nodule” in the regulation of the expression of the estrogen receptor. It remains unknown, however, whether this mechanism is equally important in all estradiol-responsive cells showing estradiol-dependent SIRT1 expression, whether this feedback is involved in both the estradiol-induced increase and decrease of SIRT1 expression, and whether one common or two separate pathways exist for ERα and ERβ. 

The above-mentioned mechanisms are described for ERβ. It is suspected that SIRT1 must also somehow be involved in the regulation of the expression of ERα because the blockade of SIRT1 action with sirtinol reduces ERα expression in MCF7 (Michigan Cancer Foundation—7) and T47D breast cancer cells; interestingly, silencing the expression of the gene encoding SIRT1 by the siRNA technique does not affect the level of receptor expression. 

SIRT1 does not bind to the promoter region of the ERα gene, nor does it affect the degree of acetylation of the histones interacting with the promoter region of this gene. How then, does the promoter activity of the gene encoding ERα become increased as a result of SIRT1 activity? It is believed that the promoter region of the gene encoding ERα is subjected to greater loading by RNA polymerase II (RNAPII) and TATA-binding protein (TBP), thus increasing its transcription due to the greater abundance of basic transcription factors; due to such increased loading, SIRT1 maintains the expression of ERα in cells (at least in the breast cancer cells with which the cited studies were performed, i.e., MCF7 and T47D), enables the expression of all estrogen-dependent genes dependent on Erα, and determines the high survival rate of breast cancer cells in vitro [51]. 

This notion sheds a light on the reasons why SIRT1 is such a pleiotropic protein involved in a plethora of cellular reactions. The cause may be its capacity to modulate transcription factors, like estrogen receptors, causal for changes in the expression of the profusion of estrogen-dependent proteins.

Some works indicate that not only classical estrogen receptors can be molecularly interconnected with SIRT1. Studies on estrogen-receptor-deficient breast cancer cells and peritumoral fibroblast cells confirmed that estradiol acts as a G-protein-coupled estrogen receptor (GPER) ligand and induces SIRT1 expression by rapid activation of the EGFR (epidermal growth factor receptor)/ERK (extracellular-signal-regulated kinase)/c-fos/AP-1 (activator protein 1) pathway. SIRT1 expression is upregulated by estradiol at the mRNA and protein levels in SkBr3 (breast cancer cells) and CAFs (cancer-associated fibroblasts). This enhancement is mediated by the GPER/EGFR/ERK factors and by the upregulation of the c-fos factor recruited to the AP-1 site in the promoter sequence of the SIRT1 gene. The activation of this pathway has clinical significance since it reduces the cytotoxicity of DNA-damaging agents, e.g., etoposide. Estradiol was found to decrease p21 (cyclin-dependent kinase inhibitor 1 or cyclin-dependent kinase-interacting protein 1) upregulation and p53 (tumor protein p53) acetylation, thereby abolishing cell cycle blockage and reducing susceptibility to apoptosis-inducing factors. Thus, SIRT1 carries the survival signal from the estradiol in mammary tumor cells [52] in a GPCR-dependent mechanism, which is different than those controlled by ERα or ERβ.

These interactions between estrogen receptors and SIRT1 explain the relationship between estradiol and the cellular lifespan, at least in cancer breast cancer cells. 

Thus, the activity of both classical and non-classical estrogen receptors stays in close and reciprocal associations with the SIRT1.

**Figure 1:**
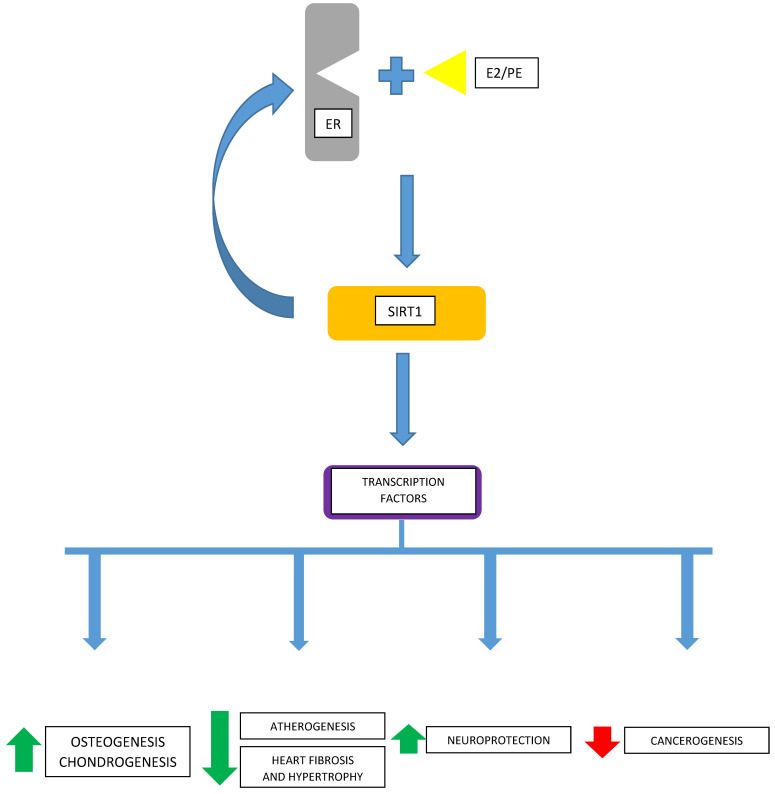
Estradiol (E2) or phytoestrogens (PEs) interact with one of the estrogen receptors (ERs) and intensify the activity of sirtuin 1 (SIRT1), which may increase the expression of the ER. SIRT1 modulates the activity of transcription factors involved in the processes regulating, among others, osteogenesis, chondrogenesis, the metabolism of molecules affecting the rate of atherogenesis, cardiac fibrosis and hypertrophy, neuroprotection, and carcinogenesis.

It remains unknown how universal these mechanisms are regarding other cancerous, or non-malignant, cells. Considering the wide expression of GPER receptors [56,57] and classical ERα- and ERβ-type estrogen receptors in both non-cancerous and neoplastic tissues, there is a need to know the ubiquity of these interactions between estradiol and cellular transcription machinery via SIRT1 and the extent of their cell-/tissue-specificity (Figure 1).

## 3. The Importance of Estradiol–Sirtuin-1 Signaling in the Physiology and Pathology of Human Tissues

The following subsections will summarize the results on the relationship between estradiol, SIRT1, and selected processes at the level of cells, tissues, and whole organisms. They will also examine these relationships in regard to certain physiological and pathophysiological processes associated with estradiol and SIRT1, which may influence the aging processes in selected human organ systems.

### 3.1. The Osteocartilage System

Osteoarthritis is the most common disease of the musculoskeletal system, characterized by the severe loss of articular cartilage, formation of osteophytes, synovial inflammation, and subchondral bone remodeling [58]. This disease progressively increases with age in men and women [59] and is known to be typical of menopause, clearly suggesting a connection with estrogen levels. Such suggestions are confirmed by studies showing that degeneration of cartilage can be reversed by estradiol and, thus, the estradiol-triggered cellular pathways may be suitable therapeutic targets for osteoarthritis [60]. 

Chondrocytes are the pivotal cells maintaining the structure and function of articular cartilage, enabling the proper distribution of mechanical forces in the joints. However, with age, chondrocytes can undergo hypertrophy, show loss of proliferation, and show a decreased ability to synthesize and secrete extracellular matrix proteins; however, they are still able to secrete matrix-degrading enzymes and proinflammatory cytokines. This imbalance is considered the key component of the pathogenesis of osteoarthritis [61,62] and, thus, chondrocytes are regarded as the main target of potential therapies.

Although human chondrocytes from osteoarthritis cartilage samples, human knee articular chondrocytes [63,64], and chondrosarcoma HTB-94 cells express SIRT1 [64], their expression decreases with age, contributing to a higher risk of osteoarthritis development in humans and mice [64,65]; this is due to a shift in chondrocyte activity toward catabolic proteolysis, increasing the rate of apoptosis, together with lower chondrogenic action [64], decreasing autophagy, and accelerating chondrocyte senescence [66]. However, the exact mechanism of SIRT1 activation in chondrocytes and its role in age-associated osteoarthritis are poorly understood. 

Estradiol could possibly be a factor affecting SIRT1 activity in chondrocytes and, thus, a factor regulating chondrocyte viability since it has been revealed that this estrogen can significantly affect these cells.

One study found that the activation of SIRT1 rescues chondrocytes from aging-associated changes through increased autophagy. The induction of autophagy in chondrocytes begins after the SIRT1-dependent deacetylation of autophagy-regulating proteins, i.e., Beclin1, ATG5 (autophagy-related protein 5), ATG7 (autophagy-related protein 7), LC3 (microtubule-associated protein 1A/1B-light chain 3) [64], paralleled by the increased expression of EGFR due to its lower ubiquitination caused by the downregulation of phosphatase and tensin homolog (PTEN) [67]. In agreement with the general view that autophagy protects cells from apoptosis, SIRT1-activating factors have also been described as antiapoptotic in chondrocytes [64]. Results showing the activation of autophagy by SIRT1 in chondrocytes come from experiments made on human cells sampled both from young [64] and older [67] volunteers.

Unfortunately, estradiol and SIRT1 are very rarely linked by researchers working on chondrogenesis.

In-vitro-obtained outcomes suggest increased SIRT1 protein and mRNA expression in chondrocytes in the presence of estradiol. Estradiol initiates a pathway in chondrocytes that leads to the activation of mitophagy, as evidenced by the greater expression of phosphorylated forms of LC3, TOM20 (translocase of outer mitochondrial membrane 20), and Hsp60 (heat shock protein 60). Increased numbers of mitophagosomes are seen in estradiol-treated chondrocytes, which indicates that the selective cellular renewal of mitochondrial components is ongoing. In addition, it is important to note that SIRT1 mediates chondrocyte estradiol signaling to AMPK (adenosine monophosphate-activated protein kinase)-phosphorylating proteins in ATDC5 chondrocytes in vitro [55] since the increased expression of AMPK and phosphorylation are often found to co-occur with SIRT1 induction in estradiol-treated cells. This is a key pathway responsible for increasing the viability of estradiol-exposed chondrocytes [55].

Thus, the estradiol-dependent and SIRT1-mediated inductions of autophagy (mitophagy in particular), associated with SIRT1-driven changes in the acetylation of autophagy-regulating factors, are responsible for antiaging effects in chondrocytes and may contribute to lower osteoarthritis morbidity.

In the elderly, disorders in the processes of bone loss and osteogenesis are both highly sensitive to hormonal factors. They appear to derive from changes in the bone marrow cytoarchitecture. The bone marrow is a source of multipotent stem cells giving birth to osteogenic or adipogenic lineages. The percentage of adipocytes in the bone marrow increases with age. It is estimated that, by adulthood, about half of the bone marrow volume is already occupied by fatty yellow bone marrow. This process is further accelerated in obesity and or in subjects on high-fat diets. The process of marrow adipogenesis shows sexual differences. In women, a rather sudden intensification is observed at about 55–65 years of age while the progression of marrow adipogenesis is slower and more gradual in men. This process results in disorders of hematopoiesis associated with the cellular and humoral impact of adipocytes on hematopoietic stem cells of the bone marrow; it is also associated with a characteristic shift of hematopoiesis towards myeloid cells, a decrease in the differentiation of stem cells into osteoblasts, and a preference for osteoclast production. These cytological variations in the bone marrow niche are reflected in reduced bone regeneration in basic conditions, especially in the case of bone damage, which often limits the mobility of older people [68,69,70].

Estradiol and SIRT1 can be signaled as factors modulating the processes of bone loss and osteogenesis. Estradiol reduces the survival and activity of osteoclasts and inhibits adipogenesis in the bone marrow. These phenomena therefore promote osteogenesis over bone loss. As osteoclast activity increases with age, the rate of bone resorption also increases. At the same time, adipogenesis in the bone marrow intensifies and begins to prevail over osteogenesis. Molecularly it can be reflected by an imbalance in the expressions of PPARγ (peroxisome proliferator-activated receptor gamma) and SIRT1. Young mice, compared to old mice, have a lesser expression of PPARγ and a greater expression of SIRT1. Ovariectomies in young and old animals demonstrate an increased expression of PPARγ and decreased expression of SIRT1, with the changes being more accentuated in the old group. Hormone replacement therapy with estradiol reverses changes in the SIRT1 and PPARγ expression induced by ovariectomies. Shifting the balance of expression between SIRT1 and PPARγ towards SIRT1 favors the differentiation of progenitor cells in the bone marrow into osteoblasts over adipocytes in mice [54], which contributes to maintaining optimal bone regeneration and self-repair and protects against age-associated disability.

The above observations confirm that the key protein for the conversion of bone marrow stem cells from osteogenesis to adipogenesis is PPARγ, especially regarding the expression of its acetylated form [70]. The inhibition of PPARγ expression, which has been reported to be achievable with the activation of SIRT1 by estradiol [54], may reduce the formation of adipogenic precursor cells in the bone marrow in mice in vivo models [54], which will reduce the number of cells that secrete osteoclastic factors, such as RANKL (Receptor Activator for Nuclear Factor κ B Ligand) [70], and promote the maintenance of the osteogenic cell population; thus, osteogenesis will potentially prevail over bone loss.

### 3.2. Cancer Cells

Cancer can be diagnosed in human subjects at any stage of life; however, its incidence increases from the age of 50. This age-dependent pattern suggests that it may be due to the accumulation of mutations inducing a highly mitotic state and resistance to apoptosis, with the further formation of tumors and possible metastasis. The molecular basis of this relationship is poorly understood, mainly due to the focus on laboratory models employing young animals and cell lines obtained from young organisms [71]. This risk may also be associated with long-term exposure to exogenous and endogenous factors that can potentially damage DNA and influence inter alia DNA repair, cell proliferation potential, histone modification, cellular senescence, and telomere attrition [1,72,73].

The role of SIRT1 in cancer is controversial. In some studies, it appears as a suppressor of cancerogenesis, in others, it appears as an activator [74].

It is not clear at all how estradiol can affect SIRT1 expression in different cancer cells. An interesting proposal has been tested using the HeLa cell line. It turned out that SIRT1 expression increases in cells after exposure to estradiol and that this results from the activation of the proteasomal degradation of the PPARγ factor, acting as a negative regulator of SIRT1 expression [75]; as such, a decrease in the concentration of the factor limiting the expression of SIRT1 in the cell obviously leads to an increase in the expression of the NAD^+^-dependent deacetylase. The NEDD4-1 (neural precursor cell expressed developmentally down-regulated protein 4-1 or E3 ubiquitin-protein ligase NEDD4-1) protein has been implicated as an E3 ligase labeling PPARγ for proteasomal degradation in response to estradiol [50]. The appearance of estradiol in the cell culture environment, or the maintenance of its physiological level in the body, most likely blocks the formation of the stable inhibitory transcription complexes of SIRT1 and PPARγ, which are assembled at the promoter sequence of the SIRT1-coding gene. 

Interestingly, SIRT1 itself is involved in regulating the activity of its inhibitor, PPARγ, as SIRT1 deacetylates PPARγ. As SIRT1 is also able to bind p300 (histone acetyltransferase p300), i.e., the PPARγ acetylase, SIRT1 can upset the balance between PPARγ acetylation and deacetylation [75].

While the increase in SIRT1 expression by estradiol appears to be a fairly universal and non-tissue-specific mechanism, as evidenced by the list of cells whose SIRT1 expression is influenced by estradiol levels, no studies have examined whether the pathways regulating SIRT1 expression and the activity related to the action of PPARγ are also not specific to cell type. Indeed, SIRT1 transcription has been found to decline with age in various animal tissues (lung, heart, fat) [75], indicating that it does not appear to be a tissue-specific response. However, the present review only regards SIRT1 expression as a select “symptom” of aging; different biochemical pathways could potentially lead to the same effect in different tissues.

It is also not known whether the factor tagging PPARγ for proteasome degradation (NEDD4-1), induced in response to estradiol, is universal in different cells or whether its role may be performed by other proteins.

Nevertheless, the promotion of PPARγ-dependent SIRT1 expression in HeLa cancer cells would certainly lead to higher cancer cell viability.

Estradiol activates a number of protein factors along the entire pathway of the lung cancer cell response to estrogen stimulation. The first protein whose expression is initiated by estradiol is ERβ. The intensification of SIRT1’s deacetylating activity in lung cancer cell lines A549 and H1435 leads to the deacetylation of the FOXO3 (forkhead box O3) factor and its increased susceptibility to ubiquitination and proteasomal degradation. This results in a decrease in the expression of the FOXO3 factor and an increase in the expression of programmed death-ligand 1 (PD-L1). The above reactions result in the stimulation of tumor growth and promotion of metastasis in mice models of lung cancer [49].

The view that SIRT1 stimulates the survival of cancer cells is additionally supported by the fact that a greater expression of SIRT1 results in higher chemoresistance and lower efficacy of chemotherapy in lung cancer cell line H292 [76]. Thus, in this regard, estradiol stimulation may increase the tumor cell viability and trigger pathways allowing cancer cells to be detoxified by chemotherapeutics. SIRT1-dependent chemotherapy failure is observed in estrogen-dependent breast cancer, in which SIRT1 increases the expression of aromatase; this may contribute to higher intra-tumor levels of estrogen and lower the clinical efficacy of aromatase inhibitors commonly used to treat breast cancer cells MDA-MB231, MCF-7, and T47D [77]. 

Greater expression of SIRT1 is positively associated with greater expression of ERα in breast cancer cells. In addition, malignant mammary cells express much more SIRT1 than the normal mammary gland epithelium due to ERα binding to the promoter region of the SIRT1 coding gene, allowing a direct interaction between them. SIRT1 not only perpetuates estrogen signaling in breast cancer cells by increasing aromatase and estrogen receptor expression but also facilitates their survival by enhancing the expression of antioxidative proteins and modulating p53 and cyclin D2 expression. Thus, breast cancer tumorigenesis and survival may be hampered by SIRT1 blockage while the enhancement of SIRT1 expression caused by estradiol may act inversely if the results obtained in the ZR75.1 breast cancer cell line can be extrapolated to other cell lines and in vivo tumors [78]. 

However, not in all cancer cells, even those commonly considered to be highly dependent on sex steroids, does estradiol have an effect on changes in SIRT1 expression. For example, in ovarian cancer cells (cell lines OVCAR3 and SKOV3), no association was observed in vitro between the exposure of cells to estradiol and the level of SIRT1 expression [79].

### 3.3. Atherogenic Metabolic Disorders and Diseases of the Cardiovascular System

SIRT1 appears to protect against cardiovascular aging by reducing inflammation and oxidative stress and by increasing the rate of autophagy; however, the exact relationship between the degree of SIRT1 expression and the cardiovascular risk/benefit ratio remains to be established [35]. Similarly, although estradiol is regarded as an antioxidative and anti-inflammatory factor in the cardiovascular system, especially during the premenopausal period, its cardioprotective efficacy and atherogenicity remain unclear [80,81]. 

A lack of estradiol in mice (due to ovariectomies completed at 5–7 weeks of age) leads to hyperplasia paralleled by the increased expression of SIRT1 in vascular smooth muscle cells in the animal aorta and supplementation with estradiol counteracted these changes. Estradiol decreased the expression of SIRT1 in these cells in vitro in a dose- and time-dependent manner, leading to lower viability, migration, proliferation, and increased apoptosis [82]. Increased apoptosis of vascular smooth muscle cells is considered to be an atherogenic factor [83] increasing plaque vulnerability [84]. Also, proliferation [85] and migration [86] are thought to be related to increased risks of atherosclerosis, restenosis, and pulmonary hypertension. The results of Lee et al. [82] suggest that the estradiol–SIRT1 axis has a detrimental effect on vasculature. 

Another model of cardiovascular aging used hypogonadism, induced by ovariectomies in aged (twenty-month-old) female apolipoprotein E knockout mice. Ovariectomies downregulated endogenous estradiol, which, in turn, downregulated SIRT1 and endothelial nitric oxide synthase (eNOS) expression in the aortic wall. At the same time, increased expressions of β-galactosidase (a marker of aging), acetylated p53, and plasminogen activator inhibitor-1 (PAI-1) were observed. Estradiol replacement therapy reversed the effects of the ovariectomies, increasing the expression of SIRT1 and eNOS and delaying vascular aging and atherosclerotic changes. The effect of estradiol was attenuated by sirtinol (inhibitor of SIRT1) or imitated by bazedoxifen—an estrogen receptor modulator. It was also shown that the overexpression of SIRT1 in endothelial cells protects against atherogenesis without changes in the glucose and lipid levels. In the endothelium, SIRT1 targets cell viability and apoptosis regulators. One of which, p53, increases the rate of endothelial senescence when acetylated. Hence, the SIRT-1-dependent deacetylation of p53 can be regarded as a senolytic action based on the maintenance of DNA integrity and efficient repair. The endothelial expression of SIRT1 is closely associated with hemostasis and the balance between vasorelaxation and contraction. At the molecular level, it is seen as an association of SIRT1, NO, and estradiol; SIRT1 expression is regulated by the NO produced by eNOS, whose expression is in turn stimulated by estradiol [42].

The discrepancies in the results obtained by Lee et al. [82] and Sasaki et al. [42] may be due to the fact that the studies were focused on two different compartments of the cell wall (smooth muscle cells versus endothelium) and, also, because ovariectomies were performed in groups of differing ages (5–7 weeks of age versus 20 weeks of age). In addition, older animals had a specific genetic background (apolipoprotein E-knockout).

Rats with streptozotocin diabetes (which serves as a model of pathological changes caused by chronic hyperglycemia, a pathological condition highly toxic to the cardiovascular system, including vascular wall components, especially vascular smooth muscle cells) demonstrated weaker SIRT1 staining in the blood vessel vascular smooth muscle cells compared to controls. Thus, it can be concluded that SIRT1 expression is reduced in the smooth muscles of blood vessels. When isolated vascular smooth muscle cells were incubated with estradiol, a decrease in SIRT1 expression was observed in control (normoglycemic) cells and there was no significant effect on the expression of SIRT1 in the vascular smooth muscle cells taken from hyperglycemic animals (where SIRT1 was already reduced in vivo). It appears that SIRT1 expression was downregulated in the vascular smooth muscle cells of normoglycemic animals by the estradiol-dependent activation of ERα, but not ERβ. Importantly, the decrease in SIRT1 expression in vascular smooth muscle cells was accompanied by an increase in the expression of AMPK, an enzyme often studied in tandem with SIRT1 as the two primary proteins regulating the cell response to changes in the energy demand of the cell [43].

It has been suggested that the decrease in SIRT1 expression observed in the blood vessel walls of animals with streptozotocin diabetes may result from a decrease in NAD+ concentration in the cells; however, SIRT1 and AMPK expression is regulated in a different way in normoglycemic vascular smooth muscle cells following exposure to estradiol in vitro [43]. This hypothesis has not yet been verified.

In vascular smooth muscle cells, hyperglycemic stress initiates SIRT1 downregulation; but, estradiol appeared to be unable to rescue and increase SIRT expression in the course of streptozotocin diabetes. It is currently unknown why estradiol added in vitro weakened the expression of SIRT1 in the smooth muscle cells of the blood vessel wall. The role played by the increase in AMPK expression observed in this process also remains unclear. It is even more difficult to reach a conclusion because the effect of hyperglycemia on the expression of SIRT1 was tested in vivo while the modulatory effect of estradiol was tested in vitro, using cells isolated from animals with streptozotocin diabetes and normoglycemic controls. Hence, the effect of lowered SIRT1 expression, induced by either hyperglycemia or estradiol, on the cell remains unclear because no studies have been performed on basic parameters, such as cell viability and their response to standard stimulating factors. 

SIRT1 and PPARγ expressions were found to decrease and increase, respectively, in the liver cells of laboratory mice following estradiol deficiency paralleled by oxidative stress in hyperoxia. Shifts in the expression of SIRT1 and PPARγ can be associated with the unfavorable, i.e., obesogenic, modulation of the metabolism and greater susceptibility of liver cells to oxidative stress [48]. Thus, we have the first evidence that maintaining high SIRT1 expression and low PPARγ expression has beneficial effects on the hepatic metabolism of glucose and lipid metabolic disorders. This has obvious significance for the prevention of cardiovascular diseases of thrombotic origin associated with unhealthy lifestyle habits (diet, physical activity). One characteristic indication of such pathologies is heart remodeling.

Cardiac fibrosis is characterized by an increase in the expression of α-smooth muscle actin (α-SMA), fibroblast-specific protein 1 (FSP1), vimentin, and TGF-β (transforming growth factor-β), along with its downstream factor Smad2/3; it is also associated with a decrease in the expression of endothelial markers (CD31, cluster of differentiation 31 or platelet endothelial cell adhesion molecules) and SIRT1. Intense collagen deposition in the interstitial myocardium has been reported in a fibrosis model in mice injected with isoproterenol. Activation of SIRT1 by resveratrol, a commonly used phytoestrogen, leads to the activation of SIRT1 and inhibition of collagen deposition in the interstitial myocardial tissue. A direct interaction was noted between SIRT1 and Smad2/3 and SIRT1 expression was reduced by TGF-β; but, this effect was effectively prevented by resveratrol. Maintaining the correct expression of SIRT1 blocks the transport of Smad2/3 to the cell nucleus in TGF-β-treated cells, which negates the profibrotic effect of TGF-β [39]. 

Overexpression of SIRT1 is a very effective way to counteract angiotensin-II-induced myocardial hypertrophy. Upregulation of SIRT1 significantly reduces cardiomyocyte size and protects the heart cells from apoptosis. An effect similar to the genetic overexpression of SIRT1 can be obtained with estradiol, which will also have a protective effect on the heart against the hypertrophic effect of angiotensin II in mice in vivo [87]. Thus, SIRT1 and estradiol can be indicated as the targets and agents of therapies treating cardiac hypertrophy and protecting against the development of heart failure associated with its remodeling.

A model of post-menopausal metabolic syndrome was created in ovariectomized animals fed with a high-fat diet. It was found that disorders in endothelial dynamics developed together with endothelial dysfunction and cardiac cellular structure alterations. The latter were associated with the increased expression of the AT1 (receptor of angiotensin) protein in the aorta and in the heart, activation of the pro-apoptotic proteins Bax (Bcl-2-associated X protein) and PARP (poly(ADP-ribose) polymerase), and increased histone H3 acetylation. These changes were reversed with estradiol replacement therapy, which partially restored normal endothelial contractility in response to angiotensin II, increased expression of SIRT1 and phosphorylated AMPK, and decreased histone H3 acetylation. The effects of estradiol were negated by sirtinol. Estradiol replacement therapy improved the functional state of the endothelium by restoring the balance between the contractility induced by angiotensin II and relaxation induced by carbachol. These effects probably resulted from the increased synthesis and bioavailability of NO (an endogenous vasodilator) due to increased eNOS expression under the influence of estradiol, SIRT1, and AMPK. In addition, estradiol inhibited the expression of angiotensin receptors, thus reducing the contractility of blood vessels. Estradiol can also protect blood vessels by inhibiting NADPH oxidase activity, thus preventing reactive oxygen species (ROS) production. Since SIRT1 and AMPK inhibitors reverse the effects of estradiol, these proteins may have a key role in shaping the described reactions; although, the authors do not position SIRT1 and AMPK in any suggested upstream–downstream relationships. But, even having no clue regarding the exact lining of SIRT1 and AMPK in one cellular pathway, we are already aware that the change in the level of acetylation of heart and vascular proteins, including histone H3, following estradiol intensification of SIRT1 activity, is the key factor. Maintaining high levels of histone acetylation is known to reduce NO synthesis and contribute to endothelial dysfunction; in addition, angiotensin II induces p300 acetylase activity following histone H3 phosphorylation. The p300 protein is also involved in the regulation of cardiac cell growth [40]. 

Changes in the expression of SIRT1 deacetylase following fluctuations in estradiol concentrations are, therefore, crucial in shaping the reactivity of the endothelium and maintaining the correct micro- and macro-vasculature of the heart. 

### 3.4. Brain

Age-related changes in the level of estradiol may also facilitate diseases of the central nervous system, including neurodegenerative and neuropsychiatric diseases [88,89]. 

SIRT1 supports the growth of axons and dendrites and helps to maintain neuronal plasticity, protecting against age-associated neurodegeneration and cognitive decline [90]. 

The potential link between estradiol- and SIRT-1-dependent neuroprotection is unclear; however, a few already-available data shed some light on this plausible association.

A neurodegeneration model induced by the chronic administration of d-galactose was investigated in male mice. Galactose induced the activation of microglia and astrocytes and the expression of phosphorylated c-Jun N-terminal kinase (JNK); it also activated the following downstream proteins responsible for neuroinflammation and neuronal apoptosis: RAGE (receptor for advanced glycation end products), NFκB (nuclear factor kappa B), tumor necrosis factor-α (TNF-α), interleukin-1β (IL-1β), COX2 (cyclooxygenase-2), p53, caspase-3, and PARP-1. The intraperitoneal administration of estradiol with galactose increased SIRT1 expression in an ERα-dependent manner in comparison to animals treated with galactose that experienced no estradiol intervention. This was accompanied by a decrease in ROS, lipid peroxidation and oxoguanosine levels, and JNK and NFκB expression, together with an increase in reduced glutathione, nuclear factor erythroid 2-related factor 2 (Nrf2), and heme oxygenase-1 (HO-1) in the mouse brains. The number of cells undergoing apoptosis and showing signs of neuroinflammation decreased under estradiol treatment. Injections of estradiol resulted in proper synapse function and improved memory in mice, better performance in the Morris water maze and Y-maze tests, more platform crossings and longer amounts of time spent in the target quadrant, and improved short-term working memory [36]. 

In addition, 17β-estradiol could improve spatial learning and memory ability. While galactose increased the activity of β-site amyloid precursor protein-cleaving enzyme-1 (BACE-1) and βamyloid protein, estradiol reversed this effect, also beneficially altering the expression of the synaptic protein synaptophysin and post-synaptic density protein 95 (PSD95) in the brain. The pivotal discovery was that estradiol-induced protection disappeared under the influence of the SIRT1 inhibitor. ERα and SIRT1 appear to play a role in the initiation and transduction of the estrogen signal in brain cells exposed to galactose and estradiol in vivo and in vitro; SIRT1 activity depends on estradiol because estradiol is able to directly bind to the N-terminal domain of SIRT1. Thus, estradiol can be regarded as a neuroprotective sirtuin-activating compound (STAC) [36].

The role of estradiol and SIRT1 in shaping the survival of neurons during aging has also been studied during the perinatal period.

One of the most common forms of neurotoxic brain damage affecting adult life is ethanol brain damage in utero, known as fetal alcohol syndrome. In general, when the developing rat brain is exposed to ethanol, adverse changes induced by triggering an inflammatory response, oxidative stress, and apoptosis occur. These changes are effectively counterbalanced or reversed by estradiol, which enhances the expression of Nrf2 and HO-1, two factors that stimulate redox-maintaining proteins. These increase the concentration of reduced glutathione and decrease oxoguanosine, a marker of oxidative DNA damage. Since estradiol helps to maintain the structural integrity of the DNA molecule, which is less exposed to ROS, it reduces the expression of pro-apoptotic proteins, such as PARP and Bax, and, consequently, the number of degenerating neurons and astrogliosis. In addition, the anti-neurodegenerative effect is enhanced by the downregulation of pro-inflammatory proteins (NFκB, iNOS (inducible nitric oxide synthase), TNF-α) and upregulation of SYP (synaptophysin) and PSD95 (postsynaptic density protein 95), as well as the downregulation of JNK, Akt (RAC(Rho family)-alpha serine/threonine-protein kinase), and mTOR (mammalian target of rapamycin) phosphorylation, which was enhanced by ethanol [37].

Ethanol strongly inhibited SIRT1 protein expression in the brains of seven-week-old rats. Co-administration of estradiol with ethanol activated SIRT1 and downregulated acetyl-p53 and phospho-NF-κB proteins, which, in turn, inhibits neurodegeneration. The authors indicate that the intermediate proteins between SIRT1 and acety-p53 and phospho-NFκB are phospho-Akt, phospho-mTOR, and phospho-JNK, whose levels are increased by ethanol but are then decreased by estradiol in postnatal rat brains (with additional confirmation in immortalized mouse hippocampal neuronal HT22 cells and BV2 microglial cells) [37].

The above results are extremely interesting but do not give much detailed information about the interactions between estradiol, SIRT, and other signaling proteins. The conclusion that estradiol-induced changes were associated with SIRT1 activation was only based on the downregulation of acetylated p53 and phosphorylated NF-κB; the analysis did not include PPAR, Bax, Nrf2, HO-1, GSH (reduced glutathione), or oxoguanosine concentrations [37], shown as being involved in fetal alcohol syndrome development; it is unclear whether their actions are also dependent on SIRT1 or on SIRT1’s downstream targets, i.e., p53 and NFκB. It would be good to examine the relationship between the particular signaling proteins occurring in the brain following ethanol and estradiol treatment, taking into account the organizing role of the estradiol-SIRT1 axis. Such studies should also include a description of the sex of the examined rat pups.

In an animal model of depression induced by 0.4 and 0.83 mg/kg b.w. lipopolysaccharide (LPS), after a 24-h observation, it was noted that old females are the most susceptible to disturbances resembling human depression. Therefore, this age–sex group was included in further research. After LPS treatment, significant decreases in serum estradiol levels were observed, accompanied by a decrease in SIRT1 mRNA and protein expression and an increase in phospho-NFκB and TNFα expression in the hippocampus and frontal cortex. Increases in the levels of Il-1β and Il-6 in the brains of the tested animals were also observed under the influence of LPS. Adverse molecular changes in the brain and changes in behavior were inhibited by the intraperitoneal administration of estradiol or an estrogen receptor agonist of ERα but not of ERβ. The results indicate that, in brains exposed to LPS, estradiol increases the expression of SIRT1, which inhibits the activation of NFκB and the synthesis of pro-inflammatory cytokines, thus resulting in an antidepressant effect [38].

Hence, it appears that the neuroprotective action of estradiol has potential clinical significance for the treatment of neurodegenerative and neuropsychiatric diseases involving SIRT1 activation.

### 3.5. Skeletal Muscles

Aging also affects the skeletal muscles. A key feature of advancing age is a loss of muscle mass, named sarcopenia, which may seriously reduce quality of life due to the loss of strength and endurance and increased frailty. The molecular basis of this phenomenon in humans is not clearly understood; but, one of the main causes is believed to be a slower rate of protein synthesis in muscles. Interestingly, loss of skeletal muscle mass can be slowed by non-pharmacological interventions, like exercises, diet, and hormonal therapies, since muscle physiology is highly dependent on androgens [91] and estrogens [92]. 

As in the case of the nervous system, transcription factors that trigger antioxidant and anti-inflammatory defenses in cells, especially Nrf2, are also activated in response to estradiol in injured skeletal muscle myocytes. 

Mechanical damage to muscle tissue was found to increase the concentration of estradiol in the circulating blood of female mice. Blood estradiol concentration peaked on the seventh day after the injury and a sharp decrease was registered on the fifteenth day. Muscle damage results in a decrease in the expression of superoxide dismutase, catalase, SIRT1, PGC1α (peroxisome proliferator-activated receptor gamma coactivator 1-alpha), Nrf2, and HO-1 in muscle tissue. Since these are factors that trigger antioxidant defense, it is not surprising that a decrease in their expression leads to an increase in oxidative damage to myocytes. However, decreases in the expression of Nrf2, PGC1α, SIRT1, and HO-1 factors can be prevented by the administration of estradiol. 

Therefore, it can be seen that muscle injury is accompanied by the induction of intense oxidative stress, one of the factors changing its expression in myocytes under such conditions is SIRT1, whose inhibition can be prevented by estradiol. In vitro studies indicated that a model oxidizing agent, i.e., hydrogen peroxide, decreases the expression of SIRT1, PGC1α, and Nrf2 in C2C12 muscle cells (a myoblast) and this effect is enhanced by the pharmacological blockade of SIRT1 with EX527 (selisistat) [41].

Thus, further important clues have been obtained showing that changes in SIRT1 expression are estradiol-dependent and related to the cellular regulation of redox balance in response to stress, including mechanical muscle damage [41].

Unfortunately, the data cited above do not refer directly nor indirectly to aging skeletal muscle and should be treated only as a hypothetical model that could potentially explain the interaction of estradiol and SIRT1 in regulating skeletal muscle function in aging organisms. They refer specifically to mechanical contusion, which may not reflect the injuries gained by muscles through years of life. This study is, however, of great importance because the significance of estradiol in shaping the ability of myoblast precursors to proliferate and differentiate and maintain the ability to contract adult muscles is poorly researched. Perhaps even more controversial is the role of SIRT1 in the response of aging muscles to stress. While SIRT1 appears to stimulate the proliferation of myoblast precursors, it simultaneously inhibits the maturation of myotubes [93]. Despite some doubts, it has been already shown that the activation of SIRT1 by resveratrol protects the tibialis anterior muscle against age-associated sarcopenia via the preservation of normal autophagic flux [94]. Estradiol, as an endogenous activator of SIRT1, may hypothetically exhibit a very similar action, especially since resveratrol and estradiol share many molecular pathways [95,96]. 

### 3.6. Peripheral Blood Mononuclear Cells

So far, we have been moving within a quite repetitive pattern in which the increase in estradiol concentration was accompanied by an increase in SIRT1 expression; although, the clinical assessment of these changes varied in a cell-specific manner. Nevertheless, the majority of the results show the following relationship: higher estradiol → increased expression of SIRT1, which leads, generally speaking, to increased cell viability (with different clinical representation and interpretation). Are there any biological systems that can exhibit some deviations from the noted pattern “higher estradiol → increased expression of SIRT1”?

Blood fluoride level influences estradiol concentration and SIRT1 expression in PBMCs (peripheral blood mononuclear cells). It was found that women with higher levels of fluoride in the blood demonstrate lower serum estradiol levels, higher SIRT1 expression in PBMCs, and lower antioxidant enzyme expression [53]. It is therefore an observation that destroys the hitherto repeatable pattern of reactions. It is not known if this is just something specific to PBMCs and to the stress of increasing the concentration of the toxic fluoride. 

This reaction is different for at least two reasons. A decrease in the expression of estradiol in the blood is accompanied by an increase in the expression of SIRT1; although, previous works suggest the opposite. Second, the increase in SIRT1 expression is accompanied by a decrease, not an increase, in the expression of antioxidant enzymes [53]. 

Interestingly, a similar phenomenon was observed in a completely different research model. A decrease in SIRT1 expression was observed under the influence of estradiol in human PBMCs isolated from the buffy coat, as well as in vascular smooth muscle cells isolated from the aorta of healthy rats; the sex of the rats was not stated but they were probably male as these are most often selected for the induction of streptozotocin diabetes. The mentioned healthy rats were used as normoglycemic controls in the experiment [43]. Thus, it appears that the direction of changes in SIRT1 expression (intensification of expression or weakening) demonstrates some tissue specificity and that species and sex may have a potential impact on the effects of some specific stressor factors.

## 4. Phytoestrogens as Modulators of SIRT1 Expression

The processes already reviewed in this paper present interesting potential for pharmacological modulation as a range of dietary and plant-derived activators are able to mimic estradiol as modulators of SIRT1 expression. This may be of particular importance in the case of estradiol-dependent extragonadal tissues susceptible to changes associated with aging [46]. In this regard, phytoestrogens deserve deeper analysis.

Sepsis is a syndrome of physiological, pathological, and biochemical disorders initiated by infection, leading to life-threatening organ dysfunction [97]. Sepsis-induced cardiomyopathy is associated with impaired myocardial circulation, direct myocardial suppression, and impaired mitochondrial metabolism [98]. The activation of the inflammatory process, resulting in increased production of pro-inflammatory cytokines, is one of the main processes leading to septic myocardial depression [98]. Septic damage to cardiomyocytes is particularly problematic because cardiomyocytes are terminally differentiated cells with very limited regenerative capacity [99]. One possible form of cardiomyocyte death during sepsis is an inflammatory programmed cell death, triggered by pathogens or inflammatory factors in the course of infection, called pyroptosis. It may be caspase-1-(classic pyroptosis) or caspase-4/5-dependent (non-classical pyroptosis) [100]. During pyroptosis, the inflammasome NLRP3 (the NLR family pyrin domain containing 3) is activated and able to recognize molecules called pathogen-associated molecular patterns (PAMPs) and damage-associated molecular patterns (DAMPs). NLRP3 activation is mainly considered to be characteristic of classical pyroptosis. This activation consists of the NFκB-dependent activation of NLRP3 transcription. NLRP3 is also activated in non-classical proptosis when caspase-4 stimulates the production of ROS in the mitochondria, which, in turn, activates NLRP3. Thus, there appears to be some overlap between the two pathways of pyroptosis (classical and non-classical) and the differences between them are perhaps most pronounced at the initiation stage: the classical pathway is triggered by microbial molecules, such as toll-like receptor ligands or endogenous molecules (e.g., TNFα), while the non-classical pathway begins with the recognition of lipid A in the LPS molecule by caspase 4/5 [101]. The consequences of sepsis-associated cell death are serious and treatments are urgently sought; estradiol and SIRT1 are investigated in this respect.

The induction of sepsis by cecal ligation and puncture causes myocardial injury and cardiac dysfunction, which can be inhibited by syringaresinol, a lignan previously described as an inducer of SIRT1 gene expression that is included in the family of phytoestrogens [102]. Syringaresinol exhibits a noticeably protective effect on cardiomyocytes both in an in vivo sepsis model and during in vitro experiments using LPS as a sepsis inducer. The cardioprotective effect of syringaresinol manifested as an increase in the expression of SIRT1 and a decrease in the activity of NLRP3, as well as a decrease in the secretion of pro-inflammatory cytokines in the septic heart. Syringaresinol attenuated LPS-induced cardiomyocyte pyroptosis in a SIRT1-dependent manner, as demonstrated by experiments using EX427 (SIRT1 inhibitor). Most importantly, it has been shown that syringaresinol is an agonist of estrogen receptors α and β and its protective, antiseptic, and anti-inflammatory effect on mice cardiomyocytes (in vivo) can be attributed to its interaction with both membrane estrogen receptors [103]. Syringaresinol has also been shown to be effective in reducing lung damage. The mechanism seems to be almost identical to the protective effect on the heart, i.e., a decrease in the expression of NLRP3 and in the phosphorylated forms of ERK, JNK and p38, as well as NFκB were also associated with the interaction between syringaresinol and estrogen receptor β [104]. Unfortunately, there is no experimental data showing that SIRT1 is involved in an estrogen-receptor-dependent rescue pathway induced in the lung by syringaresinol. It can be assumed that in the studied lung tissue [104], the anti-inflammatory effects of phytoestrogen via the estrogen receptor may be very similar to the pathway revealed in the case of myocardium [103] and in syringaresinol-exposed lungs as in the heart estrogen receptor activates SIRT1.

In both cases, NFκB factor activation was observed in response to a pro-inflammatory factor and NFκB activation was reduced by anti-inflammatory syringaresinol—a phyto-derived estrogen receptor agonist. The similarity of the trails is therefore striking. 

The molecular connection between SIRT1 and NFκB seems to be a non-tissue-specific phenomenon closely related to the regulation of the inflammatory response. Also, in other cell models, like BV2 microglia cells [105], human synovial sarcoma cells SW982 [106], and RAW264.7 (a macrophage-like Abelson leukemia virus-transformed cell line derived from BALB/c mice cells) [107], sirinagresinol was found to have an inhibitory effect on NFκB activation and to act in an estrogen-receptor-dependent manner in mice [104]. Thus, it seems that not only estradiol but also phytoestrogenic compounds have the ability to modulate the estrogen-receptor–SIRT1–NFκB axis. Importantly, this ability was observed not only in vitro [15,104,105,106,107] but also during in vivo models of acute and chronic inflammation [15,104,107], including heart [103] and lung damage [104] in the course of sepsis.

In the field of phytochemical research, it is worth mentioning that fisetin and quercetin can also increase the expression of SIRT1. In an animal model of polycystic ovary syndrome (PCOS), fisetin and quercetin induced SIRT1 expression as effectively as metformin. Both fisetin and quercetin stimulated the expression of phosphorylated AMPK in the ovaries; although, the effect was slightly weaker than those caused by metformin. These plant compounds, which have been shown to be able to upregulate SIRT1 expression in polycystic ovaries, also lowered testosterone levels and increased the estradiol blood concentration in laboratory animals treated with letrozole to induce polycystic ovary syndrome [108,109].

Unfortunately, the cited studies did not assess whether fisetin and quercetin affect the level of phosphorylated AMPK and the changes in testosterone and estradiol levels in a SIRT1-dependent manner, i.e., no attempt was made to determine whether SIRT1 is a factor “supervising” changes in the expression and phosphorylation of AMPK and steroidogenic enzymes. It has only been shown that the expression of SIRT1 changes under the action of phytoestrogens; but, the functional consequence of this change regarding the pivotal markers of PCOS was not established. Therefore, no direct molecular relationship has currently been found between SIRT1 expression, AMPK phosphorylation, and the synthesis of sex steroids in the ovaries. Nevertheless, it is highly likely that, in PCOS, SIRT1 increases AMPK expression and phosphorylation through its deacetylating activity and shifts the activity of steroidogenic enzymes towards the physiological synthesis and secretion of estradiol while blocking the signal-inducing hypertestosteronemia. Future studies should consider the potential involvement of estrogen receptors in the modulation of SIRT1 expression by quercetin and fisetin, especially since these compounds are known as phytoestrogens capable of affecting cells through estrogen receptors.

Probably the most widely studied phytoestrogen for SIRT1 activation is resveratrol. Research in this field is so extensive and advanced that it has become the subject of numerous review articles that can be easily found by interested readers.

Thus, the ER–SIRT1 axis can be modulated not only by endogenous estrogens but also by exogenous, plant-derived analogs of these sex steroids, which are already known as pharmacologically active agents in different models of human diseases. It opens new possibilities for investigations on ER-SIRT1-dependent cellular and anti-aging processes under the influence of both estradiol and phytoestrogens.

## 5. Other Nuclear Sirtuins

SIRT1, which is the focus of this review, is a member of a group of proteins that includes six other sirtuins. The entire group of sirtuins can be divided into subgroups, depending on which cellular compartment they are predominantly located in. SIRT1, along with SIRT6 and SIRT7, shows a major localization in the cell nucleus [110]. The question can be raised of whether all nuclear sirtuins show similar properties when it comes to the modulation of ER activities and whether a cross-talk of SIRT6/7–ERs may also affect the risk of age-associated diseases. Unfortunately, SIRT7 has not yet been studied in this context and we have not found a single report related to the main topic of our review.

SIRT6 appears to affect ER expression and age-related disease risk; but, the research is in the very early stages and published data are sparse.

SIRT6 stabilizes ERα expression by protecting the receptor from proteasomal degradation in the liver. This stabilization is due to SIRT6-dependent ERα deacetylation. This, in turn, leads to the activation of Fas ligand transcription, osteoclast apoptosis, and, subsequently, the reduction of bone resorption. Such a model was presented on the basis of triple lines of experimental outcomes: (a) those obtained in studies on female mice, (b) those coming from experiments on the cell culture of the HEK293T line, as well as (c) the results derived of experiments using material obtained from humans. Thus, the activation of the SIRT6-ERα pathway may be an interesting therapeutic option in age-related osteoporosis [111].

In another study, however, it has been shown that in the liver of female mice, the activation of SIRT6 reduces ERα expression and leads to a decrease in insulin sensitivity. The p300 protein plays the role of an intermediary in the signaling from SIRT6 to ERα. When SIRT6 activity is maintained in hepatocytes, estradiol cannot reveal its insulin-sensitizing properties. This leads to the development of atherogenic metabolic disorders [112]. 

Activation of SIRT6 also leads to a decrease in the expression of ERRγ (orphan nuclear receptor estrogen-related receptor γ), which leads to a reduced Cyp7a1 (cholesterol 7α-hydroxylase) transcription and, consequently, to the protection of a liver against cholestasis and fibrosis, resulting from the increased production of bile acids or their disturbed outflow. This model was proposed based on the results obtained in studies on the HEK293T cell line on murine and human liver samples [113].

Thus, the diversity of responses regulated by one sirtuin depends on the biochemical context (for example, increased exposure to bile acids) and the type of estrogen receptor involved in the SIRT6-dependent cellular pathway. In some aspects, like the impact of SIRT6 on the stability of ERα in the liver, we are still far from an unambiguous conclusion. 

Considering the different activities of nuclear sirtuins and the importance of the cellular/biochemical aspects of sirtuin actions, we should also quote interesting results of studies assessing to what extent changes in the expression of SIRT1 and SIRT6 in human breast cancer samples allow us to estimate the expected survival of patients. Here, also, the strict cellular context comes into play. In triple-negative breast cancer (no expression of estrogen receptor, progesterone receptor, and human epidermal growth factor 2 (Her2) receptor), high expression of SIRT1 is a marker of poor prognosis. In contrast, in breast cancer with positive expressions of both hormone receptors but without Her2 expression, low SIRT1 expression is a marker of poor prognosis. In the latter subtype of breast cancer, low SIRT6 expression is also a marker of a lower chance of survival. Thus, in one type of cancer, but in different histological subtypes, the expression of one type of sirtuin can either activate or inhibit carcinogenesis [114].

Such dichotomies (or even multitomies) of the influences of sirtuins on cellular processes may cause numerous problems in the designing of effective therapies that would either increase or decrease the activity of SIRT1 or SIRT6 so that they are targeted to one specific cellular pathway while simultaneously limiting the negative impact on healthy tissues.

## 6. Summary

Research on the molecular relationship between estradiol and SIRT1 expression and activity is in its infancy. Nevertheless, it appears that estradiol is indeed an important hormonal factor in modulating the activity and expression of SIRT1 in various human tissues. Of particular interest are the repeated observations showing a clinically beneficial association between the presence of estradiol in the body and a greater expression of SIRT1; this is also accompanied by the activation of metabolically beneficial molecular pathways in key tissues, such as the brain, heart, blood vessels, bones, muscles, and liver. It is also not surprising to note that the absence, or lack, of estradiol is associated with the remodeling of cellular metabolism, which impairs life quality and life expectancy and favors the development of diseases associated with aging, which can result from impaired cellular signaling governed by SIRT1. 

While the inclusion of estradiol replacement therapy appears to be a promising means of restoring SIRT1 expression and activity, at the same time, it also appears that estradiol shapes SIRT1 expression in cancer cells, which may lead to the unwanted progress of cancerogenesis and metastasis. Therefore, the cellular and tissue context is important to further research. 

It is very crucial that the relationship between estradiol and the expression and activity of SIRT1 should be viewed not only unilaterally, i.e., in a way that captures only the effect of estradiol on SIRT1, but also in a way that focuses on the effects of SIRT1 on estrogen signaling, especially the expression of estrogen receptors. 

Published studies clearly show that SIRT1 is also able to regulate its own response to estradiol or, more broadly, the estrogen sensitivity of a given cell, by acting as a modulator of estrogen receptors. Many works, discussed herein, highlight the distinction of cell-mediated effects by individual estrogen receptor subtypes (ERα versus ERβ); this tissue-specific discrimination between estrogen receptor subtypes merits further study, together with a closer look at GPCR-mediated responses, which should be also included next to classical estrogen receptors. The molecular pathways modulated by SIRT1 in response to estradiol stimulation are therefore a universal cell survival signal that is characteristic of many different tissues, both pathological and healthy, from young and old organisms. Estradiol–SIRT1 cross-talk reflects the ability of cells to adapt to metabolic and energy stress and explains the anti-aging properties of estradiol. 

The ability to control these cellular abilities through estradiol (or its analogues, e.g., phytoestrogens) and SIRT1 may help in the design of new therapeutic strategies. However, there are two key challenges. Estradiol and SIRT1 constitute a non-specific and cell-universal biochemical axis, which may impede the cell-targeted effect of future therapies; perhaps the ability to select individual pathways triggered by different estrogen receptors will be helpful. In addition, the estradiol-SIRT1 biochemical axis inhibits and activates numerous intracellular pathways, due to the fact that SIRT1 is a cellular signaling hub. 

It is currently unknown whether such non-specific effects are able to control and stimulate clinically beneficial processes. On the other hand, the potential problems of tissue and cellular non-specificity may also prove to be beneficial as they can allow several birds to be hit with one stone. The fact that the aging process is not dependent on only one molecule or pathway means the estradiol–SIRT1 pleiotropy may be beneficial; but, this makes it hard to control. Another major limitation to progression is that so much of the research on estradiol and SIRT1 is conducted in vitro, which is poorly informed regarding whole-body responses. Even so, a few studies have proven the preclinical effectiveness of modulating the estradiol–SIRT1 axis in animal models.

Some extra attention should be paid to blood vasculature since data indicate that estradiol reduces SIRT1 in vascular smooth muscle cells in vitro and demonstrates proatherogenic effects and beneficial antiatherogenic reactions in the endothelium; thus, a number of interesting discrepancies and variations remain unclear. For example, different components of the vascular wall yield different results in model studies; therefore, what clinical outcome should be expected to appear in the overall vasculature of a living organism?

Hence, the “estradiol-SIRT1” axis appears to regulate various targets, most of which are transcription factors, such as PPARγ, NFκB, and FOXO1, which, in turn, regulate mainly inflammatory reactions and metabolic/energetic switches as the pivotal cellular responses to estradiol-induced changes in SIRT1 activity. 

The studies discussed above provide an insight into the molecular basis of the association between various aspects of the aging process and changes in the functioning of the endocrine system. Demonstrating a direct link between estradiol and SIRT1 activity and the cellular viability of the brain, cardiovascular system, and bones sheds new light on the issue and indicates pathways towards future anti-aging strategies.
